# Wagging the long tail of drivers of prostate cancer

**DOI:** 10.1371/journal.pgen.1007820

**Published:** 2019-01-17

**Authors:** Vincent L. Cannataro, Jeffrey P. Townsend

**Affiliations:** 1 Department of Biostatistics, Yale University, New Haven, Connecticut, United States of America; 2 Program in Computational Biology and Bioinformatics, Yale University, New Haven, Connecticut, United States of America; 3 Department of Ecology and Evolutionary Biology, Yale University, New Haven, Connecticut, United States of America; Dana Farber Cancer Institute, UNITED STATES

Armenia and colleagues [1] recently analyzed the largest set of prostate cancer exomes to date—1,013 exomes from 680 primary and 333 metastatic tumors. Whereas it has been suggested that gene discovery is near saturation for localized nonindolent prostate cancer [2], Armenia and colleagues demonstrated the utility of uniformly analyzing data sets from a large number of clinically diverse tumors from the same tissue. Through their analysis, they revealed 97 significantly mutated genes and found that the prevalences of these mutated genes follows a long tail, with a few genes containing a substitution in comparatively many tumors, and many genes containing a substitution in few tumors. This “long tail” distribution of significantly mutated genes suggests that increases in sample size still lead to the discovery of rarely mutated but significant drivers. Indeed, the long-tail distribution of potential drivers gives hope in our fight against cancer, increasing the number of genes and pathways that might be productively therapeutically targeted by precision medicine.

The imagery of the long tail elicits not just the idea that in each cancer type there continue to be genes that affect tumorigenesis and cancer development that can still be discovered by continued sequencing. It also inherently evokes the relative ranking of those genes and their importance in explaining cancer. Armenia and colleagues follow a well-developed decade-long tradition of analysis of tumor sequence data to identify drivers—a tradition that has used two ways to describe this long tail. One way, exemplified by Lawrence and colleagues [3], ranks members of the tail of cancer genes discovered by their *P* value. However, if one’s goal is to uncover and propose the relative contribution of genes implicated in cancer to the cancer phenotype (i.e., genetic alterations contributing to increased cellular division or cellular lifetime), then *P* values are not an appropriate metric, because *P* values are thresholds of belief and not measures of effect [4].

Armenia and colleagues follow a second approach that is also common: ranking genes discovered by the prevalence of the mutations that are observed at high frequencies (because of the number of cells in a typical cancer, all mutations observed at statistically significant allele frequencies via high-throughput sequencing are present within a large number of cells within the cancer; otherwise they would be exceedingly unlikely to be observed even with deep sequencing). Ranking genes by prevalence makes more sense than ranking by *P* value. Given statistical significance, genes that are most prevalent are those that affect tumorigenesis and cancer development in the greatest number of patients. Therefore the relative prevalence of mutations observed at high frequencies within tumors (the long tail reported by Armenia and colleagues) usefully conveys how many patients might benefit from a therapeutic that targets the mutant state of the gene.

There is now, however, a third way to rank drivers of a cancer type based on mutations observed at high frequencies in tumor sequence data, a way that perhaps has been in the back of the mind of scientists all along as they have thought about cancer drivers. They can now be ranked by their relative contribution to survival and proliferation of the cancer; i.e., the intensity by which the mutated lineages are naturally selected to expand and reach detectable levels in the neoplasm and/or tumor. This measure compares the actual flux of substitutions observed to the expected flux of substitutions in the absence of selection and is the equivalent of the “scaled selection coefficient” or “selection intensity” used in population genetic research [5–7]. Selection intensity conveys how much a patient might benefit from a targeted therapeutic that fully abrogated the gain-of-function associated with an oncogenic mutation.

Further analysis of the extensive data gathered by Armenia and colleagues on prostate cancer, estimating selection intensity on specific single nucleotide mutations [5,6], and ranking observed mutations by selection intensity yields a long tail of cancer driver mutations with a very similar overall shape to that seen when ranking genes by prevalence as in Armenia and colleagues (Fig 1; S1 Table). However, ranking observed mutations by selection intensity provides a markedly discrepant ranking of the drivers. This discrepancy in rank is the consequence of enormous variation of mutation rate (among both genes and trinucleotides) and mutational target size (tumor suppressors usually can be disabled in many ways, but proto-oncogenes require very specific mutations to become oncogenes). Genes such as *AR* and *KMT2C*, with comparatively high mutation rates and many different oncogenic mutations present within tumor samples, have a lower selection intensity for specific single nucleotide variants and therefore a lower comparative ranking versus a ranking by the total number of substitutions within the gene among tumors samples. Other genes, such as *CUL3* and *BRAF*, have relatively low mutation rates and feature recurrent substitutions of one specific amino acid change, and therefore the selection for these variants must be comparatively high in order for us to observe these mutations at detectable levels within tissue from numerous tumors.

**Fig 1:**
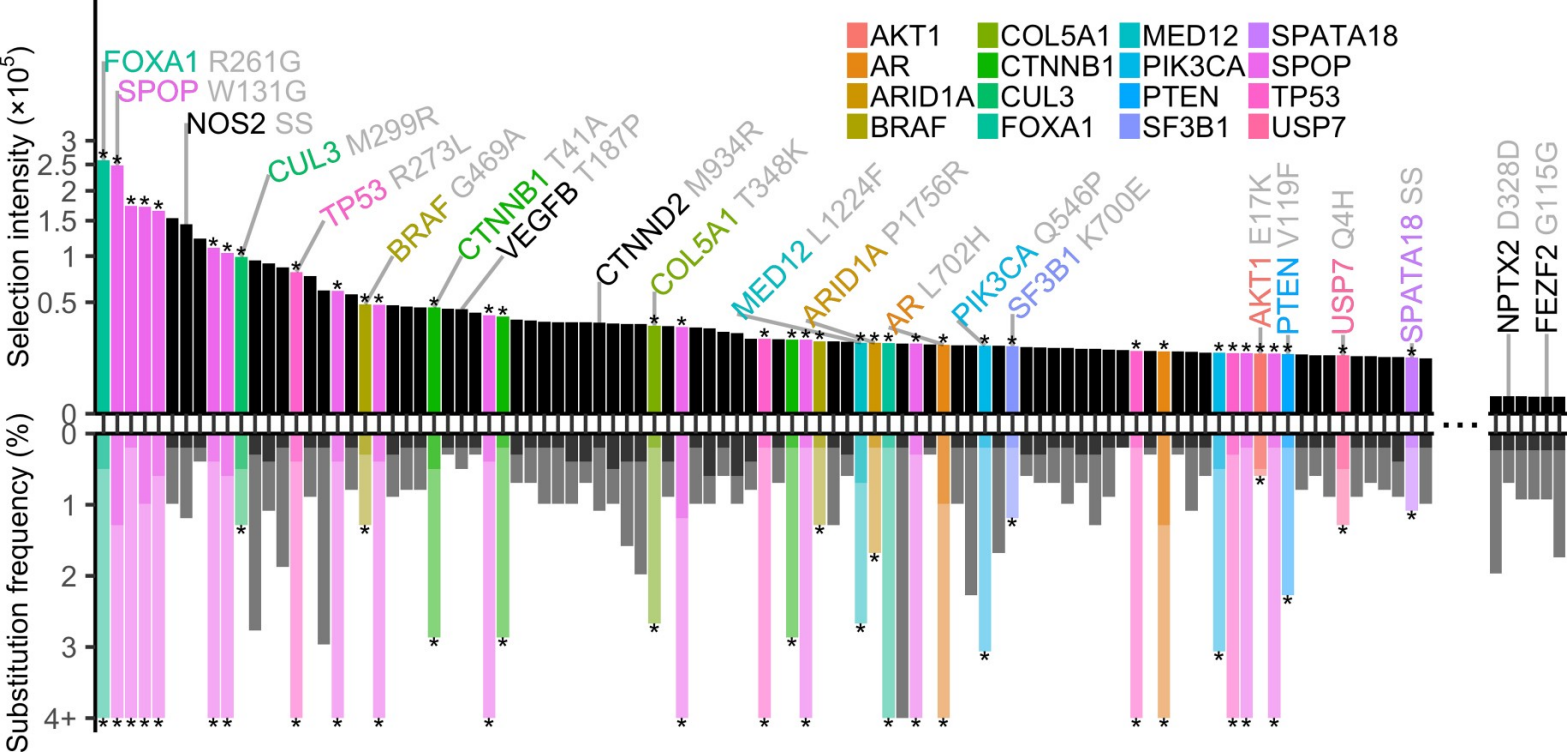
Selection intensity and substitution frequency of the 97 recurrent single nucleotide variants out of the 890 available in the Armenia and colleagues’ data set with the highest selection intensity and the 6 with the lowest selection intensity (all 6 are synonymous substitutions). Bars above the *x*-axis convey the selection intensity (plotted here on a square-root scale), with significantly mutated genes (as designated by Armenia and colleagues) labeled with an asterisk (*) and plotted each with a unique color. Bars below the *x*-axis convey the prevalence of the mutation at the gene level (semitransparent), overlaid with the prevalence of the specific single nucleotide variant ranked (solid). The NOS2 SS label refers to a substitution at chromosome 17, nucleotide position 26087772, and the SPATA18 SS label refers to a substitution at chromosome 4, nucleotide position 52946086 (hg19 coordinates). SS, splice site.

Armenia and colleagues are prescient about the importance of the novel cancer drivers that they discuss, which appear in their long tail at low prevalence but which turn out to be genes that have high impact on tumorigenesis and cancer development. Recurrently substituted sites in *CTNNB1*, *CUL3*, *BRAF*, *ARID1A*, and *SF3B1* described within Armenia and colleagues are within the top selection intensities calculated (Fig 1). Several recurrently mutated single nucleotide variants with previously described associations with prostate cancer and/or metastatic phenotypes, such as *NOS2* [8], *CTNND2* [9], and *VEGFB* [10], were not found to be significantly mutated at the gene level yet nevertheless are estimated to have remarkably high selection intensities, illustrating that there is much yet to learn about the potential translationally relevant genetic basis of some prostate cancers with additional tumor sequencing and even larger sample sizes.

How is it that the genes that Armenia and colleagues picked out to discuss, among many from far down their long tail in prevalence, would turn out to be genes of high selection intensity? Presumably, Armenia and colleagues chose to highlight newly statistically significant genes in the long tail for which—despite their low prevalence—there was known molecular biology supporting a strong and clear role in cancer. The frequent elevation of these mutations to detectably high cancer-cell counts in tumors (on the infrequent occasions when the mutations occur in a suitable cancer stem-cell lineage) indicated by intensity of selection validates the inference Armenia and colleagues made that they are genes with clear and important roles in the cancers that carry those mutations. Reciprocally, we would argue that it also points out how useful it is to quantify the intensity of selection within cancer lineages, and how useful it is to “wag” the long tail of oncogenic drivers—ranking the genes by their cancer effect size rather than by their *P* value or their prevalence.

## Supporting information

S1 Table Mutation rates, selection intensities, and prevalences for sustitutions. (TXT)
